# Intraspecific Sensory Diversity and the Decapod Claw: Patterns of Sensillation Are Heterochelic and Sexually Dimorphic In *Pagurus bernhardus*

**DOI:** 10.1002/jmor.70054

**Published:** 2025-05-13

**Authors:** Ari Drummond, Tianna Holloway, Summer Nash, Alexander D. M. Wilson, Lucy M. Turner, Mark Briffa, David T. Bilton

**Affiliations:** ^1^ School of Marine and Biological Sciences University of Plymouth Plymouth Devon UK; ^2^ University of Plymouth, MBERC Plymouth Devon UK; ^3^ Department of Zoology University of Johannesburg Johannesburg Auckland Park South Africa

**Keywords:** chela, crustacea, hermit crab, scanning electron microscopy (SEM), sensilla, sensory morphology, sexual dimorphism

## Abstract

Information detection affects physiological performance and behaviour and is vital to survival and fitness. Despite the recognised importance of sensory adaptations in information acquisition and manipulation, many forms of sensory variation—from within individuals to between species—remain underexplored. To better understand the role of information in evolution, it is important to examine sensory variation as part of a cohesive framework of sensory diversity. Using the decapod claw, a structure well‐recognised for its morphological variation, we investigated sensory diversity at the intraspecific level by assessing heterochely and sexual dimorphism in the chelar morphologies of *Pagurus bernhardus* hermit crabs. We employed a novel methodology using scanning electron microscopy (SEM) to assess moulted chelar tissue from both the major and minor claws. The shape, size, and sensillation (i.e., the distribution and abundance of sensilla) of both chelipeds were examined by geometric morphometric landmark analysis (GMLA), generalised Procrustes analysis (GPA), and linear mixed effects models. Hermit crabs exhibited heterochely and sexual dimorphism in both gross and sensory chelar morphologies. Sexual dimorphism was greater in the sensory morphology of the major claw, suggesting sex‐based sensory specialisations, likely due to differences in mating roles and behaviours. In contrast, the minor claw's sensory morphology lacked sexual dimorphism, suggesting the sensory role of this appendage is equally important for both sexes. Our results highlight sensory variation as a fundamental aspect of functional morphology and emphasise the need to consider sexual dimorphism and body asymmetry in information acquisition. These findings contribute to a broader framework for studying sensory diversity, underscoring the importance of integrating sensory morphology, function, and ecology to fully understand the evolutionary implications of sensory specialisations.

## Graphical abstract

Information detection is crucial for biological life, yet many aspects of sensory diversity remain underexplored. Using *Pagurus bernhardus* hermit crabs, we examined intraspecific sensory diversity in chelar morphology. Examinations of scanning electron micrographs of chelar moults by geometric morphometric analyses and linear mixed modelling revealed sexual dimorphism and heterochely in both gross and sensory chelar morphologies. Our findings highlight the role of sensory variation in functional morphology and its evolutionary significance in information acquisition.

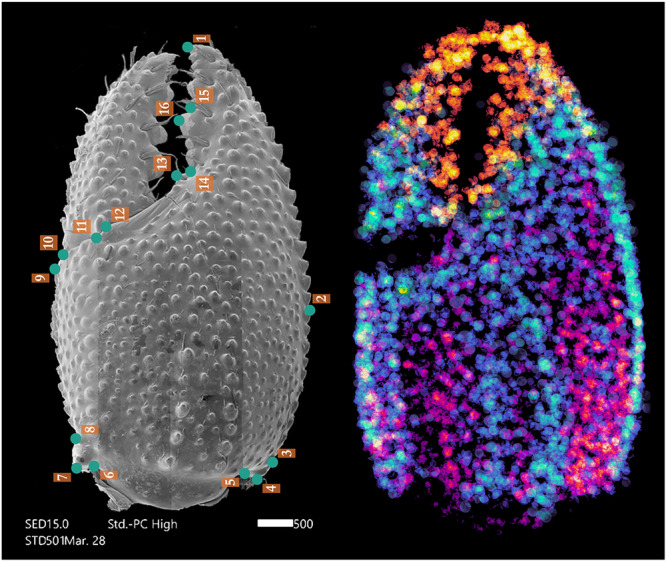

## 1 Introduction

Sensory diversity exists across hierarchical scales, from the molecular (e.g., receptor) to the organismal level. Research from information ecology, sensory biology, and morphology has demonstrated the importance of sensory diversity in information acquisition and manipulation and shows how sensory structures and processes contribute to evolutionary trajectories and adaptation (Dall et al. 2005; Derby and Thiel 2014; Schmidt et al. 2010; Stevens 2010; Watling and Thiel 2013). Within a given species, individuals may exhibit variability due to morphological plasticity and asymmetry (Kelley et al. 2018; Meng et al. 2012; Riddle and Purves 1995). The sensory arsenal of individuals, in terms of structure and performance, can change during ontogeny, through experience, or in response to the environment (Kelley et al. 2018; Lisney et al. 2007; Roth et al. 1992). Furthermore, conspecifics in a population may exhibit different sensory phenotypes based on sex, experience, or reproductive status (Hurtado et al. 2015; Mogdans 2019; Tiarks et al. 2024; Vincent et al. 2005). Despite such wide‐ranging sources of intraspecific sensory diversity, many levels and sources of this important form of biological variation remain underexplored.

Here, we aim to examine the intraspecific sensory diversity of hermit crab chelipeds (claws), structures recognised for high degrees of morphological diversity and sexual dimorphism (Claverie and Smith 2010; Hamasaki and Dan 2022; Ismail 2021; Lee 1995; Nogueira et al. 2022; Shimoda et al. 2005; Trevisan et al. 2012; Santos and Trevisan 2012; Yamaguchi 2001; Yasuda et al. 2017). Research on chelar sensory morphology in decapod crustaceans is limited (Altner et al. 1983; Zielinski et al. 2008; Belanger and Moore 2009; Hamilton et al. 1985; Moore and Belanger 2006), and chelar sensory structures have yet to be thoroughly described in hermit crabs (but see Mesce 1993a). Chelipeds are used in resource acquisition, defence, mating behaviour and communication (Fujiwara and Kawai 2016; Mariappan et al. 2000). In decapods, they are calcified structures, often featuring associated sensory capacities through the presence of pores, denticulations, and sensilla (Garm et al. 2013; Mariappan et al. 2000; Mesce 1993a). At the interspecific level, there is conspicuous variation in the function and performance of these structures, with chelae exhibiting a wide range of specialisations (Claverie and Smith 2007). Despite this variation, there appears to be a conservation of two well‐recognised chelar forms that correspond with foraging technique (Mariappan et al. 2000; Vermeij 1977). “Crusher” claws tend to be larger and more robust with molariform denticulation, whilst elongate “cutter” or “shredder” claws feature sharper, serrate denticulation (Mariappan et al. 2000). Both forms may be present in a species characterised by chelar asymmetry or heterochely. Whilst such variation in claw shape has been used as a metric of functional specialisation (Behrens Yamada and G. Boulding 1998), such considerations have not extended to incorporate sensory capabilities.

In addition to functional heterochely, decapod chelipeds exhibit sexual dimorphism in claw size and shape, driven by sexual selection (Azofeifa‐Solano et al. 2020; Correa and Thiel 2003; Lee 1995). One of the best examples are the fiddler crabs (e.g., the Ocypodidae), where chelipeds are used as weapons or signals of quality in competition for mates (Callander et al. 2013; Cothran 2020). Whilst sexual dimorphism in the gross morphology of anatomical structures, including chelipeds, is well‐described, potential sexual dimorphism in the sensory components of such structures is rarely assessed (but see Hallberg et al. 1997; Johansson and Hallberg 1992). Given sexual dimorphism and asymmetry in chelar gross form and function, one would expect accompanying chelar sensory specialisations to be an important component of intraspecific sensory diversity in decapods.

We aim to broaden the examination of heterochely and sexual dimorphism from gross chelar morphology to encompass sensory structures. Using the common hermit crab *Pagurus bernhardus*, we examine differences in the size and shape of the major (MJC) and minor (MNC) chelipeds, compare these differences between females and males and determine whether variations in the presence and distribution of sensory structures are components of heterochely and sexual dimorphism. Whilst many types of structures contribute to the sensory capabilities of chelae, we focus on sensilla and their infracuticular articulations (Garm 2004), as these structures are abundant on both claws in *P. bernhardus*.

Patterns of sensillar abundance and distribution (i.e., sensillation) may vary due to the functional requirements of the claws and the whole organism. Heterochelic but not sexually dimorphic patterns of sensillation would suggest that claws are similarly specialised in both sexes. The absence of sensory heterochely in the presence of gross structural heterochely would indicate that even though the claws are functionally different (i.e., crusher vs. shredder), their sensory capabilities may be comparable. However, differing patterns of sensillation between chelar types would suggest sensory specialisation of the crusher (MJC) and shredder (MNC) claws, indicating that these structures perform different roles in information acquisition. For example, greater sensillation of the MJC may point to prioritisation of information related to weaponry and defence, whilst greater sensillation of the MNC could indicate prioritisation of information related to the fine‐scale manipulation of food items. Sexual dimorphism without sensory heterochely would suggest that females and males are specialised in sensory capabilities, likely due to different mating roles or behaviours. For example, if males have greater sensillation overall, they may be more capable of gathering information than females, possibly due to the need to find and guard mates in the complex and variable environment characteristic of the rocky intertidal. A finding of both sensory heterochely and sexual dimorphism in *P. bernhardus* would indicate that claws are specialised to gather information according to different sex‐based priorities. As morphology may change with ontogeny and sexual maturity, we used hermit crabs from a wide mass range (a proxy for age in this species) to examine whether heterochely and sexual dimorphism of sensillation become more prominent as crabs grow.

## 2 Materials and Methods

### 2.1 Animal Collection and Maintenance

*Pagurus bernhardus* (Linnaeus, 1758) hermit crabs (N_female_ = 34, N_male_ = 42) were collected from Hannafore Point, Cornwall, UK (50°20'34.4“N 4°27'07.3“W; N_female_ = 17, N_male_ = 19), and Mount Batten, Devon, UK (50°21'20.2“N 4°07'38.2“W; N_female_ = 17, N_male_ = 23) between May 2023 and January 2024 (Linné and Salvius 1758). Whilst we could have removed limbs by induced autotomy (Walus et al. 2023), given increasing concerns regarding decapod welfare and sentience (Birch et al. 2021; Crump et al. 2022; Elwood 2025; Souza Valente 2022), we determined to devise a novel, noninvasive methodology to examine chelar morphology and sensillar topology. Hermit crabs grow by moulting with both chelipeds moulted simultaneously. As exuviae maintain the key features of the body surface (see below), we decided to use the chelar moults of each crab in place of ablated limbs. Whilst this extended the overall length of our study, it allowed us to use a greater number of individuals and develop a protocol for examining decapod species that accommodates increased concerns regarding decapod welfare.

Hermit crabs were individually maintained until moulting in a temperature‐controlled laboratory (15°C ± 1.5°C) in a 1‐l tank filled with filtered, aerated seawater (salinity: mean = 34, range = 32–37) with biweekly water changes. All crabs were checked daily and fed every 3 days on a combination of marine pellets (Vitalis Aquatic Nutrition, World Feeds Ltd., UK) and macroalgae (e.g., *Palmaria palmata* or *Saccharina latissima*). Housing in isolation ensured the crabs remained in good condition and reduced the likelihood of pre‐moult damage to chelae. Before isolation, hermit crabs were extracted from their gastropod shells using a bench vice by carefully cracking the shell along a line of fracture and removing shell debris by rinsing the crabs with seawater. Crabs were placed in a folded cotton towel for 30 s to remove excess water, after which the crab mass was measured using a bench‐top balance (OHAUS STX223, OHAUS Europe, Switzerland; precision: ± 0.001 g). We used hermit crabs from a wide mass range (mean mass: 0.508 ± 0.257, range: 0.015–1.088) to examine the relationships between mass, chelar size, and sensillation and, further, to explore whether these effects differ by sex or claw type (MJC vs. MNC). Sex was determined using a Leica EZ4 stereomicroscope (Leica Microsystems, Germany) by examining both pleopods and gonopores. Upon placement in the housing unit, crabs were provided with a *Littorina littorea* gastropod shell within ±10% of the predicted optimal shell mass (OSM), determined by the equation: *OSM* = *3.601 x crab mass* + *0.502*. All individuals were monitored twice daily until moulting, at which time tissue was collected and prepared for analysis.

### 2.2 Tissue Collection and SEM Sample Preparation and Imaging

Whilst scanning electron microscopy (SEM) has been used to investigate fine‐scale morphological variation in crustaceans (Cavey et al. 1992; Pohle and Telford 1981; Salazar and Brooks 2012; Sandberg 1970; Shelton et al. 1975; Vittori et al. 2018; Williams 2007; Wortham and LaVelle 2016), SEM techniques generally require the sacrifice of the animal or ablation of the appendage under examination (Akhter et al. 2015; Chandran et al. 2016; Sandberg 1970; Weisbaum and Lavalli 2004). Our novel method for examining chelar structure and sensory topology using SEM and image analysis of moulted tissue eliminates the necessity of death or damage. This technique was verified by comparing the chelar topology of hermit crabs that had either dropped limbs through autotomy or died during laboratory acclimation with the chelar moults of laboratory‐housed animals. We found no differences between the two tissue types in either gross or sensillar morphology. As moults clearly preserved structural morphology, we determined that moults could be used in place of freshly preserved tissue in the morphometric analysis of chelipeds and chelar structures.

**Figure 1:**
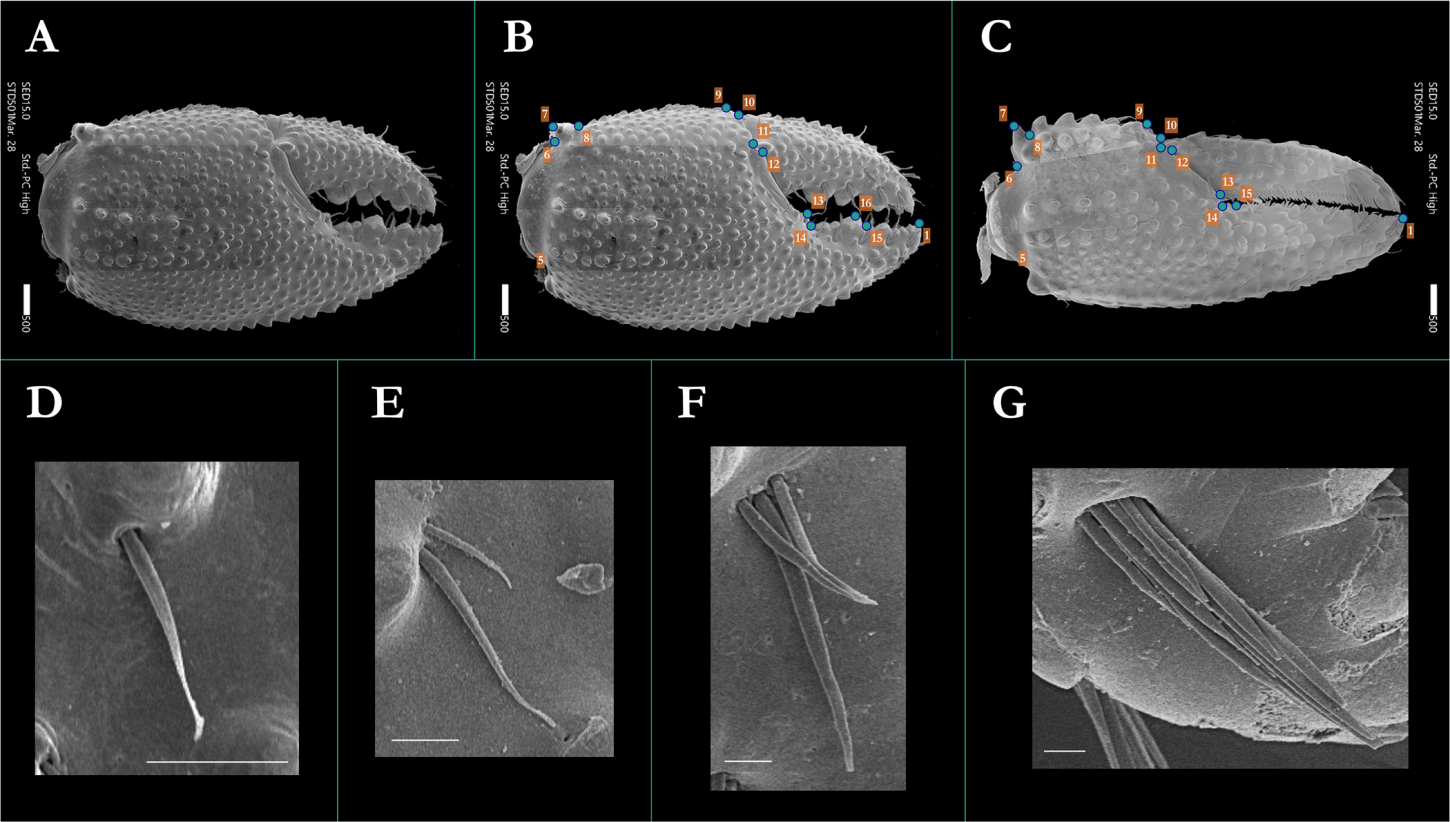
(A) Scanning electron micrograph montage image of the dorsal surface of the major cheliped (MJC) of *Pagurus bernhardus*; scale bar = 500 μm with the localisation of assigned landmarks (B) LM1 to LM16 for the dorsal surface of the MJC and (C) LM1 to LM15 for the minor cheliped (MNC). Cropped and zoomed micrographs showing (D) single (S1), (E) double (S2), (F) triple (S3) and (G) clustered (S4) articulation sites; scale bar (white) = 50 μm.

The major (MJC) and minor (MNC) chelipeds were separated from the rest of the moult at the carpal‐propodal joint and placed in a 3 cm petri dish. Salt, bacteria, and debris were removed by rinsing each chelar moult three times with deionised (DI) water and twice with 70% ethanol. The dish was then covered and left to air dry. Once dried, the chelar moult was mounted dorsal (lateral) side up on a metal stub (Agar Scientific Ltd.) topped with an adhesive carbon tab (Agar Scientific Ltd.). Tissue‐mounted stubs were gold sputter‐coated using an Emitech K550 Gold Sputter Coating Unit (Quorum Technologies Ltd., UK). All samples were analysed using a JEOL JSM‐IT510LV (JEOL Ltd., Japan) scanning electron microscope (SEM) under high vacuum using secondary electron imaging (SEI) at variable magnification determined by claw size (100 – 130X). Micrographs of chelar shape and topology were assembled by montage imaging (Figure 1A). Montage scanning was set to automatically adjust imaging parameters (focus, contrast, brightness, and alignment), with final alignment of SEM images manually corrected where necessary. We obtained a total of N = 76 MJC micrographs and N = 56 MNC micrographs, with the discrepancy in sample number attributed to the more fragile nature of the MNC.

### 2.3 Assessment of Chelar Size and Sensillar Site and Density

Micrographs of chelar structures were pre‐processed for morphometric analysis by standardising orientation and enhancing feature contrast in ImageJ (Schneider 2012; v1.53k). The chelar area (mm^2^) was assessed by obtaining an outline of each claw, scaled with the micrograph scale bar. All chelipeds were measured for both the total chelar area (including the dactylus) and the propodus only (comprising the manus and propodal extension). The morphology of *P. bernhardus* chelar sensilla is characterised by a long, undifferentiated shaft (i.e., lacking ornamentation), with infracuticular articulation and without a discernible terminal pore in intact hairs. Thus, all chelar sensilla appear consistent with the definition of simple sensilla (Garm 2004; Hallberg et al. 1997; Hallberg and Skog 2010; Wroblewska 2002). There were no clear morphological traits by which we could distinguish between chelar simple sensilla, except for variable length and articulation groupings. Four grouping patterns were identifiable: single (1 setal articulation), double (2 setal articulations within < 1 setal diameter, or approximately 20 μm of one another), triple (3 such articulations), and clustered ( ≥ 4 such articulations, homologous to the feature described by Mesce (1993a) for *P. hirsutiusculus*); hereafter S1, S2, S3, and S4 (Figure 1D‐G). We were unable to extract the exact numbers of sensilla within an S4 site due to the projection and compression of a 3D structure to 2D micrographs. Whilst Garm (2004) defined simple sensilla as having a length‐to‐diameter ratio of > 15, in our specimens, this ratio was extremely variable within articulation groupings and between S1 and all other grouping types. Given this high degree of variability, we focused on the abundance and distribution of articulation groupings (i.e., S1 to S4) rather than attempting to subdivide sensilla into simple sensilla subtypes based on the length‐to‐diameter ratio.

To assess sensillar distribution, we examined scanning electron micrographs of S1 to S4 articulation sites and marked each type using a multi‐point counter to identify both site number and placement. Sensillar site density, an indicator of sensory investment by sensillar abundance, was calculated for each type (S1 to S4) by dividing the number of sensillar sites by the total chelar area (sites/mm^²^). However, sites occupy unequal areas, showing a general pattern where the area of S1 < S2 < S3 < S4. Thus, hermit crabs may vary in the chelar surface area occupied by sensillar articulations or in sensory investment by sensillar coverage. To determine if hermit crabs vary in this latter form of sensory investment, we determined the weighted sensillar density by calculating the average area of each site type and normalising this average by dividing the focal site area by the average area of S1 types (e.g., average area of S4/average area of S1) and multiplying these values by their respective type counts (i.e., the values used in calculating sensillar abundance). This resulted in four weighted counts, one for each sensillar type, all weighted by sensillar site surface area (or coverage). These weighted counts were summed and divided by the total chelar area (see supplementary online material for a worked example). Both measures of sensory investment are valuable for assessing sensory diversity. While sensillar abundance provides a useful and easily obtained standard for examining chelar sensillation, sensillar coverage offers an estimate of how concentrated sensory capabilities are on the chelar surface.

Frequency maps of sensillar site distribution for chelae were generated by standardising micrograph alignment and size (5162 ×2947 pixels). Images were adjusted by subtracting the background and increasing the contrast to enhance feature identification of each site type. Individual sensillar site maps were thresholded to remove background features, converted to binary image files, and aggregated into image stacks. For each stack, a Z projection was obtained by summing slices to display feature locations, to which the ‘Gem’ lookup table (LUT) filter was applied. Composite maps of sensillar site density by type were created to show sensory heterochely and sexual dimorphism. Sensillar abundance frequency maps were created by merging the four site‐specific maps into a single image (frequency of site localisations irrespective of type). Sensory investment by coverage (site‐specific localisation frequency) maps were created using a distinct LUT (colour‐specific) for each site type (S1: magenta, S2: cyan, S3: lime green, and S4: gold) before merging the site‐specific frequency maps.

### 2.4 Geometric Morphometric Landmark Analysis

Three TPS files were generated in TPSUtil32 (Rohlf 2015) from the micrographs: one for MJC scans, one for MNC scans, and one comprised of a combined set of all chelar scans. A total of N = 16 MJC (Figure 1B) and N = 15 MNC landmarks (Figure 1C) were identified based on prominent propodal features. Identified landmarks (Table 1) included those homologous with chelar landmarks from past studies of decapod crustaceans (Alencar et al. 2014; Candiotto et al. 2023; Claverie and Smith 2007; Nascimento et al. 2024; Trevisan et al. 2012), with additional landmark assignment of protrusions strongly featured on the proximal portion of the chela of *P. bernhardus*. We initially included landmarks for features of the dactylus; however, subtle shifts in its position and orientation during SEM preparation and imaging introduced an unacceptable degree of measurement error; thus, only propodal landmarks were used in the final analyses. To assess heterochely, the MNC was reflected along the y‐axis for orientation alignment of MJC and MNC landmarks. MJC‐LM14, a landmark unique to the MJC propodal extension, was removed from the combined sample set to ensure equal landmark numbers for the combined analysis. All landmarks were identified and digitised using TPSDig (Rohlf 2015). Landmark assignment was performed three times by a single researcher, with randomisation of images, and verified by a second researcher.

**Table 1:** Description of landmarks used to assess shape‐based heterochely and sexual dimorphism in *Pagurus bernhardus*.

Landmark	Description
LM1	Distal tip of the propodal extension
LM2	Inferior‐most point defining the base of the curve of the inferior chelar margin
LM3	Inferior point of emergence of the inferior marginal tubercle
LM4	Maximum point defining the apex of the curvature of the inferior marginal tubercle
LM5	Superior point of emergence of the inferior marginal tubercle
LM6	Inferior point of emergence of the superior marginal tubercle
LM7	Maximum point defining the apex of the curvature of the superior marginal tubercle
LM8	Superior point of emergence of the superior marginal tubercle
LM9	Distal point of the superior manus margin
LM10	Superior junction of the propodus and the dactylus along the superior chelar margin
LM11	Superior indentation of the propodus along the margin of the border between the dactylus and the propodus
LM12	Superior protrusion of the propodus along the margin of the border between the dactylus and the propodus
LM13	Inferior protrusion of the propodus at the base of the margin that forms the border of the articulation between the dactylus and the propodus
LM14	Inferior indentation of the propodus at the base of the margin that forms the border of the articulation between the dactylus and the propodus
LM15	Point at which the superior margin of the propodal extension becomes approximately level with LM1. In the MJC, generally concurrent with the distal base of the largest molariform tooth (LM16); in the MNC, usually coincident with the proximal‐most emergence of shredding (i.e., any sharp, angular, and serrated projection) denticulation
LM16	MJC‐only, apex of the curve of the largest molariform tooth, generally occurring midway along the line of the superior margin of the propodal extension

### 2.5 Data Analyses

Shape‐based analyses were conducted using MorphoJ software (Klingenberg 2011). Landmark configuration was first standardised and optimised before assessment by generalised Procrustes analysis (GPA; Rohlf and Slice 1990), with crab ID included as a random effect (to account for two measures taken per individual, one from each claw), chelar type (MJC vs MNC) as a fixed effect (to assess heterochely), and sex as a fixed effect (to assess sexual dimorphism). General Procrustes analysis was run for the pooled data set of 15 homologous chelar landmarks and on each claw separately. Canonical variance analyses (CVA) with permutation tests were conducted to evaluate the degree of heterochely and sexual dimorphism in chelar shape, along with any sex‐based differences in heterochely. Due to missing landmark data, some scans were excluded from the analysis, resulting in N = 70 MJC, N = 54 MNC, and N = 124 scans in the pooled sample set. Wireframe plots were generated in MorphoJ to help visualise shape‐based heterochely and sexual dimorphism.

Size‐ and sensory‐based heterochely and sexual dimorphism were assessed in R (version 4.4.1; R Core Team 2024) using a linear mixed effects model (LMEM), with the log‐transformed chelar area (of the manus and propodus only for consistency with geometric morphometric landmark analyses) as the outcome variable, and log‐transformed crab mass, crab sex, and chelar type as predictor variables, with crab ID included as a random intercept (to account for the two measures of chelar area from each crab, one from each chelar type: MJC vs. MNC).

Potential dimorphism in chelar sensillation was also examined by LMEM. We modelled patterns of sensillation for absolute and weighted sensillar site densities and the proportions of each site type (focal type/total site number) as outcome variables, with crab mass, crab sex, and chelar type as predictor variables and crab ID included as a random effect. LMEMs were estimated with restricted maximum likelihood (REML) estimation by Satterthwaite's method with the ‘lme4’ (version 1.1‐35.5; Bates et al. 2015) and ‘lmtest’ (version 3.1‐3; Kuznetsova et al. 2017) packages. We began with saturated models with stepwise removal of nonsignificant interaction effects irrelevant to our key hypotheses (i.e., sexual dimorphism and heterochely). Models were assessed visually by examining residual plots and with the check_model() function in the ‘performance’ package (version 0.12.2; Lüdecke et al. 2021). All statistical figures were generated with ‘ggplot2’ (version 3.5.1; Wickham 2016). As crabs were collected from two different field sites (due to a temporary regulatory restriction during our study), we examined the potential effect of the collection site on morphological variation for all analyses. However, as the site was not significant in any analysis, it was removed as a factor, and hermit crabs in this study were treated as a single population.

## 3 Results

### 3.1 Shape‐based Heterochely and Sexual Dimorphism

**Figure 2:**
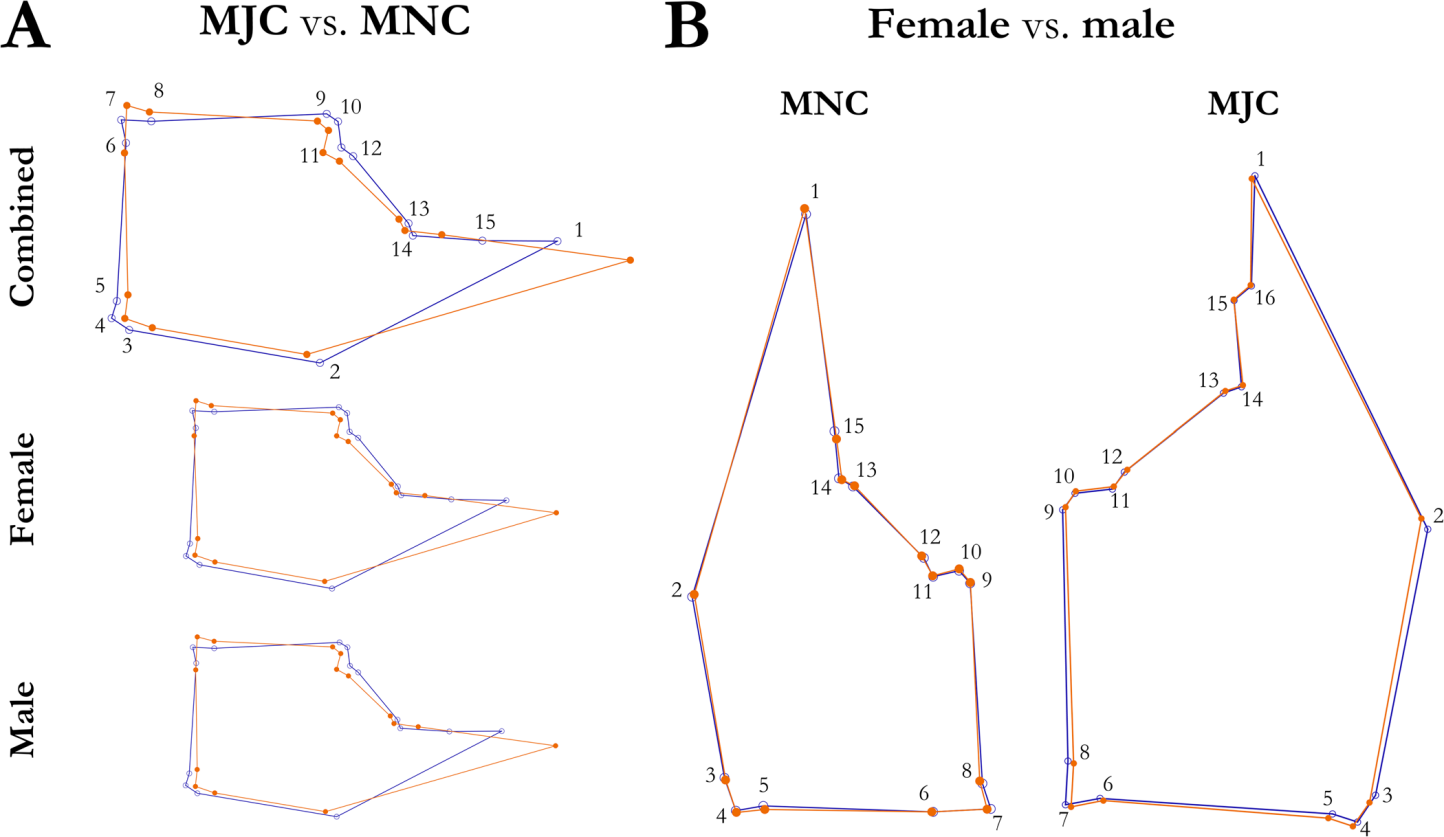
Wireframe diagrams showing shape‐based (A) heterochely and (B) sexual dimorphism in the major (MJC) and minor (MNC) chelipeds of *Pagurus bernhardus* hermit crabs. In (A), the differences between the MJC (blue) and MNC (orange) are derived from the pooled data set, the results of which are presented for males and females combined and separately. The MNC has been reflected along the Y axis to show the orientation of landmarks in its correct anatomical orientation relative to the midline of the hermit crab. In (B), chelar sexual dimorphism is shown for female crabs (blue) and male crabs (orange), based on the individual datasets for the MJC and MNC.

The shape of the MJC was significantly different from that of the MNC, with near‐perfect separation of features (F = 172.41, df = 26, Pillai trace = 0.99, *p* < 0.0001; Figure 2A). There was also a significant difference in MJC and MNC centroid size (F = 11.94, df = 1, *p* = 0.0013). The MJC was relatively stouter and wider than the MNC, whilst the MNC was longer, especially at the propodal extension. GPA also revealed significant shape‐based differences between male and female crabs for the pooled data set (F = 1.92, df = 26, *p* = 0.0037, Pillai trace = 0.71), although there was no sexual dimorphism present in centroid size (F = 0.34, df = 1, *p* = 0.56). While in both sexes, patterns of heterochely were similar (i.e., a rounder MJC and longer MNC), CVA with permutation tests revealed clear separation of both claws and sexes and significant sex‐based differences in heterochely (Goodall's F = 77.0652, *p* < 0.0001; Pillai's trace = 1.5323, *p* < 0.0001). Female crabs showed less shape‐based heterochely (Procrustes distance = 0.15, Mahalanobis distance = 11.74) than male crabs (Procrustes distance = 0.16, Mahalanobis distance = 12.41). Individual crabs significantly differed in their claw shapes (F = 1.15, df= 1066, *p* = 0.012, Pillai trace = 13.63), although not in centroid size (F = 1.15, df = 41, *p* = 0.33). For the MJC‐only data set, there was a significant difference in chelar shape between male and female crabs (F = 3.61, df = 1, Pillai trace = 0.60, *p* = 0.012; Figure 2B). There was no difference in MJC centroid size between sexes (F = 0.48, df = 1, *p* = 0.49). In contrast, for the MNC‐only data set, there was no significant difference in shape (F = 1.06, df = 1, Pillai trace = 0.50, *p* = 0.47; Figure 2B) or centroid size (F = 1.6, df = 1, *p* = 0.21) between male and female crabs.

### 3.2 Size‐based Heterochely and Sexual Dimorphism

**Figure 3:**
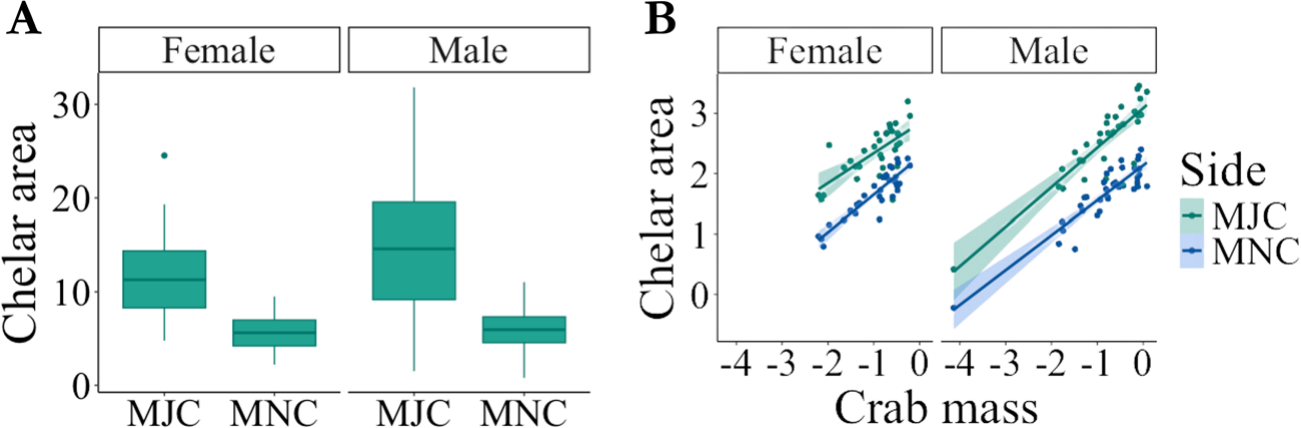
A. Size‐based heterochely in terms of total chelar area (mm^2^) in male and female *Pagurus bernhardus* for both the major (MJC) and minor (MNC) chelipeds. B. Relationship between log crab mass (log(g)) and log chelar area (log(mm^2^)) for male and female hermit crabs for both claws.

Chelar area (mm^2^) was significantly greater in the MJC than MNC (F_1,65_ = 126.71, *p* < 0.0001). Male crabs had larger MJCs than female crabs, and size‐based heterochely was greater in males than females (F_1,65_ = 5.41, *p* = 0.023; Figure 3A). There was a significant positive relationship between crab mass and chelar area (F_1,63_ = 238.92, *p* < 0.0001; Figure 3B). However, there was no interaction between crab mass and crab sex (F_1,63_ = 0.55, *p* = 0.46) or between crab mass and chelar type (MJC vs MNC; F_1,64_ = 0.036, *p* = 0.85) on chelar area.

### 3.3 Sensory Morphology and Chelar Dimorphism

**Figure 4:**
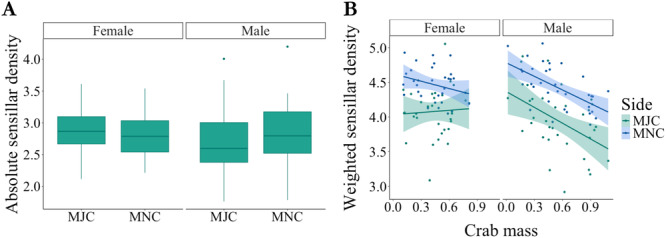
A. Sensory investment in terms of log‐transformed absolute sensillar site density (sites/total chelar area in mm^2^) on the major (MJC) and minor (MNC) chelipeds of female and male *Pagurus bernhardus*. B. Relationship between crab mass (g) and sensory investment by coverage (i.e., log‐transformed weighted sensillar density) for both the MJC and MNC in male and female hermit crabs.

**Figure 5:**
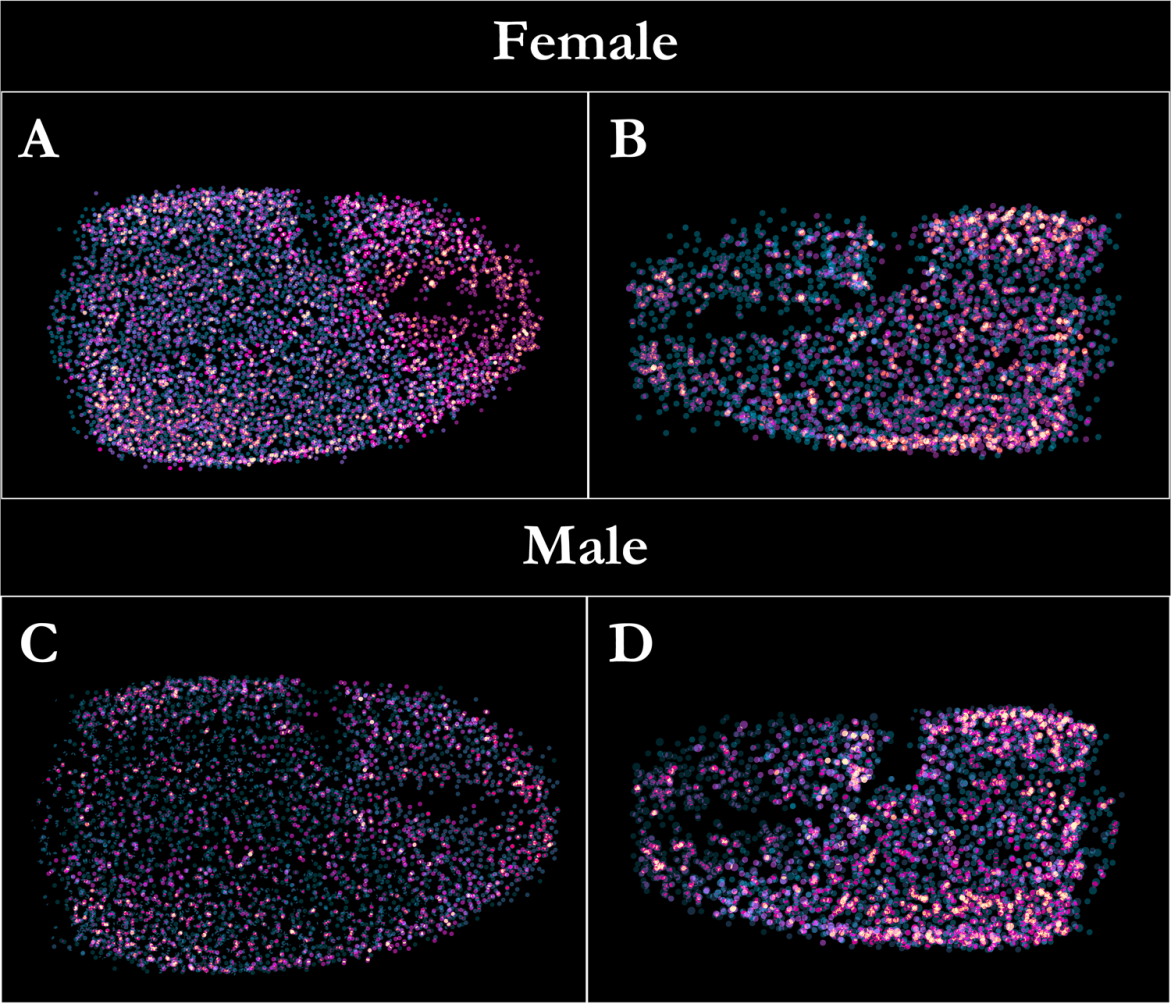
Frequency maps showing the absolute sensillar abundance (i.e., the number of sensillar articulation sites) on the dorsal chelar surface in female (A = MJC, B = MNC) and male (C = MJC, D = MNC) *Pagurus bernhardus* hermit crabs. Brighter colouration indicates a greater frequency of sensillar articulation.

A significant interaction effect between sex and chelar type on absolute sensillar site density revealed that female crabs had a higher MJC sensillar density than males (F_1,58_ = 5.17, *p* = 0.027; Figure 4A). Males, however, had a greater absolute density on the MNC compared with the MJC (Figure 5A‐D). Additionally, females had greater weighted sensillar site density than males overall (F_1,59_ = 4.19, *p* = 0.045). Absolute sensillar site density decreased with increasing crab mass (F_1,58_ = 116.84, *p* < 0.0001); however, there was no difference between males and females (F_1,58_ = 0.37, *p* = 0.54) or chelar type (F_1,58_ = 0.015, *p* = 0.90) in this relationship. There was also an overall decline in weighted sensillar site density with crab mass (F_1,58_ = 8.42, *p* = 0.0052). A nonsignificant interaction effect between crab mass and sex showed that whilst the weighted sensillar site density declined with increasing mass in males, this change was not significant in females (F_1,58_ = 3.47, *p* = 0.068; Figure 4B). Although there was no significant interaction effect between sex and chelar type on weighted sensillar site density (F_1,61_ = 0.84, *p* = 0.36), weighted sensillar site density was significantly greater for the MNC than the MJC (F_1,61_ = 67.17, *p* < 0.0001; Figure 4B).

**Figure 6:**
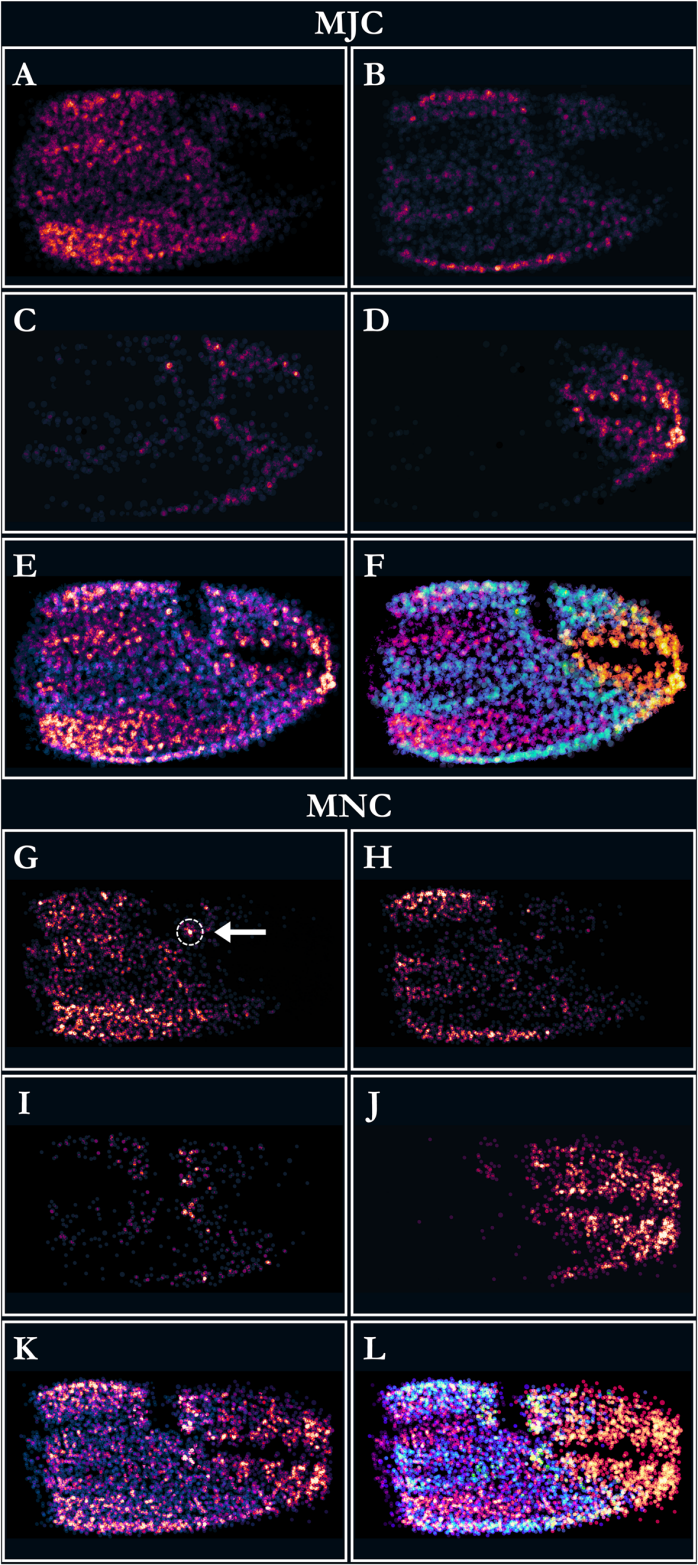
Frequency maps showing the distribution of sensillar sites by type of articulation grouping for the major (MJC) and minor (MNC) chelipeds of *Pagurus bernhardus*. For the MJC, single (S1) sites (A) were most abundant proximally; double (S2) sites (B) were concentrated at the chelar margins; triple (S3) sites (C) were sparse and located distally; and clustered (S4) sites (D) were located at the tips of the dactylus and propodal extension. A combined frequency map (E) shows site distribution on the MJC dorsal chelar surface. In (F), this map is coded by colour with the frequency of S1 (magenta), S2 (cyan), S3 (lime), and S4 sites (gold) delineated. For the MNC, S1 sites (G) were most abundant proximally and inferiorly; S2 sites (H) were concentrated at the chelar margins, especially superiorly, and the midline; S3 sites (I) were more abundant than on the MJC and were found most frequently around the propodal‐dactyl joint; and S4 sites (J) were heavily featured at the tips of the dactylus and propodal extension, more so than on the MJC. The combined frequency map (K) shows the site distribution on the MNC dorsal chelar surface. In (L), this map is coded by colour with the frequency of S1 (magenta), S2 (cyan), S3 (lime), and S4 sites (gold) delineated. The arrow and dotted circle in (G) show the location of the unique single sensillar (S1) site present on all MNCs examined.

Sensillar site density and the types of articulation groupings varied across the dorsal chelar surfaces, and the number of sensillar site types varied between individuals (σ = 0.031, SD = 0.17, ICC = 0.41; Figure 6E,F,K,L). On the MJC, S1 sites were most abundant, being most common proximal to the carpal‐propodal joint and diminishing distally, with additional areas of higher density at the superior and inferior margins of the claw and on the lower third of the claw, from the inferior margin, to a heavily tubercled ridge of the MJC propodus (Figure 6A). S2 sites were less abundant on the MJC and were concentrated primarily at the superior and inferior margins of the propodus, with two additional regions of higher density, approximately parallel on either side of the chelar midline (Figure 6B). S3 sites were least common on the MJC, being concentrated distally along the perimeter of the dactylus and propodal extension (Figure 6C). S4 sites were concentrated distally on the MJC, especially at the tips of the dactylus and propodal extension (Figure 6D).

As with the MJC, MNC sensillar sites were concentrated distally along the propodus and dactylus and along the chelar margins, both superiorly and inferiorly. S1 sites were abundant across the MNC propodus, especially proximal to the carpal joint, with a region of high density along the inferior margin (Figure 6G). A unique single sensillar site was identified as a homologous structure on the dactylus of the MNC of all crabs examined. This sensilla was localised along the midline of the dactylus, proximal to the propodal‐dactyl joint (Figure 6G). Whilst visually similar to all other chelar sensilla at the magnification used, the site was distinguished by its much longer sensillum, with a deeply recessed articulation. A similar site was sometimes featured on the MJC, but without the same consistency in localisation or form. As on the MJC, MNC S2 sites were abundant posteriorly, especially at the superior and inferior margins and proximal to the carpal joint (Figure 6H). MNC S3 sites were more prevalent than those on the MJC and were found around the propodal‐dactyl joint (Figure 6I). S4 sites were heavily featured on the distal portions of both the dactylus and propodal extension (Figure 6J).

The proportion of S1 sites was greater on the MJC than on the MNC (F_1,62_ = 629.67, *p* < 0.0001), whilst the proportions of S2 (F_1,64_ = 76.05, *p* < 0.0001), S3 (F_1,125_ = 19.33, *p* < 0.0001) and S4 sites (F_1,65_ = 456.55, *p* < 0.0001) were all greater on the MNC than the MJC. There was a significant effect of mass on the proportions of all sensillar sites (S1: F_1,63_ = 26.93, *p* < 0.0001; S2: F_1,63_ = 10.24, *p* = 0.0022; S3: F_1,125_ = 9.10, *p* = 0.0031; S4: F_1,64_ = 9.38, *p* = 0.0032) and the correlation was positive for all types except S1 sites, where proportion decreased as crab mass increased. There was no significant effect of sex and no significant interaction between sex and chelar type on the proportion of any sensillar site type; however, there was a nonsignificant trend for females to have a higher proportion of clustered sensilla (S4 sites) than males (F_1,65_ = 3.13, *p* = 0.081).

## 4 Discussion

*Pagurus bernhardus* chelipeds exhibit sexual dimorphism and heterochely, with significant differences evident in both gross and sensory morphology. Whilst minor chelar (MNC) traits are similar between male and female crabs, the major cheliped (MJC) is sexually dimorphic. Male MJCs are larger and more robust, whilst female MJCs have a greater absolute sensillar site density than male MJCs. Furthermore, females have higher weighted sensillar densities on both claws. In terms of gross structural heterochely, the MNC is smaller and more elongate than the MJC. Regarding sensory heterochely, the MNC weighted sensillar site density (i.e., sensillar coverage) exceeds that of the MJC, and the claws differ significantly in the proportion of sensillar site types, with single (S1) sites predominating on the MJC and grouped (S2, S3, and S4) types being more proportionately abundant on the MNC.

### 4.1 Chelar Sexual Dimorphism

In terms of gross morphology, although we did not observe significant sexual dimorphism in the MNC, we found pronounced sexual dimorphism in shape, size, and sensillation in the MJC. This may suggest that the use of and information gathered by this claw differs between females and males. Shape‐based sexual dimorphism of the major claw has been demonstrated in snapping shrimp (Nascimento et al. 2025) and fiddler crabs (Hartnoll 1974). In these species, major chelae function as weapons or signalling tools, with sexual selection influencing the degree of dimorphism present. In *Alpheus* shrimp, selection favours males with larger, more robust chelipeds as males use their larger claw in both fighting and signalling (Nascimento et al. 2025). Meanwhile, sexual dimorphism in the major chela of *Cinetorhynchus* shrimp has been linked to mate‐guarding behaviour, with larger males having greater reproductive success (Bauer et al. 2014). It is noteworthy here that *P. bernhardus* also mate‐guard (Lancaster 1988). Although the importance of chelar morphology in determining reproductive success has not been examined in this species, in the related *P. nigrofascia*, males with larger, more robust chelipeds are better able to acquire and defend potential mates from competitors (Yasuda et al. 2011).

In terms of sensory capacity, we found that females exhibited greater sensory investment in both absolute and weighted sensillar density. The female MJC displayed higher levels of sensillation than males, and, although not statistically significant, females tended to have a greater proportion of S4 sites. S4 sites are most common along the distal tips of the propodus and dactylus, the chelar regions most likely to be used in detecting and manipulating resources, such as food and shells. Thus, females may possess an enhanced ability to identify and select between necessary resources. Improved access to environmental information may increase the rate of resource acquisition (i.e., food) needed to sustain higher reproductive output. Additionally, as an egg‐brooding species, female *P. bernhardus* may require more detailed information regarding viable empty gastropod shells. Hermit crabs use their chelipeds to assess shell size and quality (Elwood and Stewart 1985; Mesce 1993b). Shell fit is known to influence fecundity in female hermit crabs, and reproductive females must select shells with architecture adequate to accommodate a growing mass of eggs (Bertness 1981; Elwood et al. 1995). A greater sensory capacity, especially of the grasping regions, could help females to better assess shell resources and refine selection during shell investigations.

Alternatively, the observed sensory sexual dimorphism may partly result from allometric scaling. Female *P. bernhardus* are smaller in overall body size than male conspecifics (Schmidt et al. 2024). Smaller crabs have smaller claws with less surface area available for sensory input. However, this mass‐area relationship is unlikely to be the sole reason for sensory sexual dimorphism, given that the relationship between mass and weighted sensillar density is quite distinct between female and male crabs (Figure 4A). With increasing crab mass (a proxy for age in this species), the weighted sensillar density generally decreases, except for the female MJC. As crabs grow, the sensory investment by coverage in female crabs largely remains constant. Given that the absolute density still decreases with crab mass, this result suggests that females may increase the size of sensillar clusters or articulation sites as the claw grows. As suggested above, females may use enhanced sensory abilities to support reproduction and resource acquisition. Considering that brood size tends to increase with crab age, such increased sensory abilities could enhance fitness and fecundity.

Furthermore, although it is to be expected that crab and claw size are positively correlated, our finding that crab size and sensillar site density are negatively correlated in males and in the female MNC is surprising and contradicts research on antennular sensilla, or aesthetascs, which shows increasing sensillar abundance with age (Beltz et al. 2003). It is possible then that total chelar sensillar abundance changes little over the lifespan of the crab (i.e., the number and proportion of sensillar remain relatively constant as the claw grows), and thus become more dispersed over the surface as chelar size increases, leading to reduced density. Crabs may develop sensory specialisations during ontogeny, likely as a form of sensory plasticity in response to experience or environmental change. To date, this form of sensory plasticity has not been investigated in hermit crabs; however, plasticity and developmental specialisations of sensory and neuronal structures have been reported in other species (Anton and Rössler 2021; Johnson 1988; Polley et al. 2008; Raevsky et al. 1997). For example, antennal sensillation have been shown to change in response to diet in grasshoppers (Bernays and Chapman 1998) and to climate in bees (Boulton and Field 2022). It is likely that hermit crab sensillation also changes in response to the environment and during ontogeny. Future studies could examine if and how different biotic and abiotic variables impact sensillation and sensory investment in a wide diversity of invertebrate sensory appendages to improve understanding of the plasticity of information acquisition and its impacts on survival and fitness.

### 4.2 Heterochely

Our findings of heterochely at different scales show that *P. bernhardus* claws are specialised in both gross and sensory morphology. Thus, chelipeds are functionally specialised in terms of their foraging and resource manipulation capabilities (e.g., gastropod shell assessment) and in information acquisition and sensory capabilities. Furthermore, these traits are likely linked. As in many decapods, the *P. bernhardus* MJC is a larger, more robust ‘crusher’ claw, whilst the elongate MNC has a characteristic ‘shredder’ form. Both claws are heavily sensillated; however, the greater weighted sensillar density of the MNC likely relates to its primary role as a foraging tool. The MNC surface is characterised by exceptionally high sensillar density at the distal tip and medial margins of the dactylus and propodus, regions likely important in food detection and handling. Whilst a higher proportion of grouped sensilla, especially at the chelar tips, may aid in foraging and the fine‐scale detection and discrimination of resources, the higher weighted sensillar density across the MNC dorsal surface suggests that this appendage may be more specialised for information acquisition than the MJC. Such a conclusion is further supported by the lack of sexual dimorphism in the MNC.

In contrast, observed patterns of MJC sensillation may relate to how this appendage is used as a weapon and as a facultative ‘operculum’. *Pagurus bernhardus* hermit crabs are well known for their agonistic interactions in pursuit of shell resources, utilising their chelipeds as signals and weapons (Briffa 2013). As a weapon, the MJC would benefit from high sensory input in and along the ‘pinch zone’. This is an area with high sensory capacity in both claws. However, whilst in the MNC, this region may be useful for foraging, in the MJC, this region may assist in fighting. In the giant rhinoceros beetle *Trypoxylus dichotomus*, sensillar density on the horn is concentrated in regions most frequently used during fighting (McCullough and Zinna 2013). Thus, high sensillation of this region in hermit crabs may help in ‘shell war’ tactics (Dowds and Elwood 1983). Meanwhile, the overall greater proportion of S1 sites on the MJC may be associated with signal detection during hermit crab retraction. When a hermit crab retracts into its gastropod shell, its chelipeds act together as a quasi‐operculum, occluding the shell aperture whilst remaining exposed to the surrounding media. Of the two chelae, the larger, more robust MJC occupies the bulk of this space, at times overlapping the MNC. The S1 sites on the MJC dorsal surface may detect chemical or mechanical signals pertaining to environmental threats, including predators (*in review*). A higher proportion of S1 sensilla, particularly on the more exposed MJC, could assist in threat detection and reflect investment in a risk‐mitigation strategy. Whilst all sensillar groupings likely function in signal detection during retraction to some extent, the observed heterochely in the proportion of S1 sites suggests that these hairs might be primarily responsible for environmental monitoring during retraction, a prospect that merits further investigation.

### 4.3 Chelar Sensilla and Articulation Clustering

In both claws, all sensilla appear to be simple sensilla, with putative bimodal chemo‐mechanosensory function irrespective of articulation grouping type (S1 to S4). The sensory modality of these chelar single sensilla remains uncertain. However, the socket‐like infracuticular articulation of each hair suggests at least a mechanoreceptive function (Garm 2004; Hallberg and Hansson 1999; Hallberg and Skog 2010; Laverack and Barrientos 1985; Watling and Thiel 2013), although bimodal chemo‐mechanoreception has also been suggested (Mesce 1993a). Whilst the type of information gathered by distinct sensillar morphotypes has been examined (Derby and Weissburg 2014; Garm et al. 2013), the effects of sensillar grouping and articulation density have not yet been assessed. Although morphologically homologous to the simple sensilla described in other species (Garm 2004), there may be an underlying functional specialisation relating to sensillar grouping and distribution in *P. berhnardus*. One possibility is that S1 sensilla are mechanoreceptive, whilst the sensilla comprising S2 to S4 sites are bimodal or functionally partitioned (i.e., some of the sensilla within the grouping are mechanoreceptive and others chemoreceptive). Additionally, grouping sensilla could be a way to protect crabs’ information‐detection capabilities through a form of functional redundancy, as has been observed in other sensory structures (Knowlton and Gaffin 2011). During SEM imaging, we observed that sensilla are frequently damaged by loss, abrasion, fraying, or breakage. Such damage to a sensillum could render the structure inoperable, requiring a moult to regain sensory function. Thus, grouping sensilla could be a way to offset costs of damage, especially in areas frequently in contact with environmental surfaces and resources, such as the chelar tips and margins. Indeed, these are the areas where S2 to S4 sites are most frequent. Future research could investigate the effects that sensillar grouping and articulation density have on sensory performance through a combination of electrophysiological and behavioural studies and ultrastructural analyses.

## 5 Conclusion

The findings in this study provide strong evidence that sensillation, an important component of the functional specialisations of decapod chelipeds, is a sexually dimorphic and heterochelic trait in *P. bernhardus*. This form of sensory diversity is a key feature of intraspecific variation with functional consequences for individual fitness. Whilst studies have described gross morphological differences in chelar form and function, especially between species, few have considered these structures as part of an integrative framework that incorporates sensory diversity within a single species. Here, we link the importance of intraspecific sensory diversity to functional capabilities, placing them in the context of wider chelar functional traits and the different selection pressures on female and male hermit crabs due to sexual selection. Information acquisition and transmission are fundamental to all living systems. To better understand how life evolves and specialises in acquiring, generating and transferring information, it is essential to explore sensory diversity at all levels within a cohesive and integrative framework, bridging gaps in our understanding across scales and systems.

## Author Contributions

**Ari Drummond:** conceptualization, investigation, writing – original draft, methodology, visualization, writing – review and editing, formal analysis, project administration, data curation, supervision. **Tianna Holloway:** Data curation, visualization. **Summer Nash:** data curation, validation. **Alexander D M Wilson:** supervision, writing – review and editing. **Lucy M Turner:** writing – review and editing, supervision. **Mark Briffa:** supervision, writing – review and editing. **David T Bilton:** writing – review and editing, supervision, methodology.

## Ethics Statement

Institutional ethical approval was not required when the present project was initiated, and *P. bernhardus* hermit crabs are currently an unregulated invertebrate species. However, we followed the ASAB/ABS Guidelines for the ethical treatment of nonhuman animals in behavioural research and teaching (ASAB Ethical Committee/ABS Animal Care Committee 2023). No hermit crabs were intentionally harmed during the experiment. Furthermore, we ensured that the space, food, and living conditions provided during laboratory housing were of the highest quality available. Additionally, all hermit crabs were returned to their collection site as soon as possible, generally within 2 weeks of collection.

### 1 Peer Review

The peer review history for this article is available at https://www.webofscience.com/api/gateway/wos/peer-review/10.1002/jmor.70054.

## Supporting information

Supplementary Information.

GMLA data.

SensoryHeterochely.

Chelar R Script.

## Data Availability

The data that supports the findings of this study are available in the supporting material of this article.
