# Supplementary material for: Intraspecific Sensory Diversity and the Decapod Claw: Patterns of Sensillation Are Heterochelic and Sexually Dimorphic In Pagurus bernhardus
Source: J Morphol. 2025 May 13;286(5):e70054. doi: 10.1002/jmor.70054 (PMC12075039; doi:10.1002/jmor.70054)
Supplement: Supplementary file 1 — Supplementary Information. [file JMOR-286-e70054-s003.docx]

**Title:** Intraspecific sensory diversity: Patterns of sensillation are heterochelic and sexual dimorphic in *Pagurus bernhardus*

**Supplementary Information**

**Weighted sensillar density, a worked example**

Count data was obtained for all sensillar articulation site types and chelar area as described in the methods. These data were used to obtain the total sensillar site density for each cheliped (Table S1).

| **Table S1:** Sensillar site counts and absolute sensillar density | | | | | | |
| --- | --- | --- | --- | --- | --- | --- |
| **Site type counts** | | | | | **Chelar area**  (mm^2^) | **Total sensillar site density**  (total sites / mm^2^) |
| **Single** | **Double** | **Triple** | **Bundled** | **Total** |  |  |
| 125 | 48 | 9 | 18 | 200 | 17.4612698 | 11.4539207 |

After obtaining absolute counts and to the absolute site density (sites/mm^2^), the weighted site density was determined by finding the proportional area of each site type relative to the average area of a single site (Table S2). The proportional area was multiplied by the site count for each site type. The sum of these weighted counts gave us a metric of the proportional sensory investment, relative to the investment in a single sensilla, for each cheliped examined. This total (e.g., 710.93, Table S2, below) was divided by the total chelar area (e.g., 17.46, Table S1, above), giving us the weighted sensillar site density (e.g. 40.71, in this example).

| **Table S2**: Site type average areas (mm^2^) and proportional areas, a ratio, used to obtain the individual and total site count weighted by area | | | | |
| --- | --- | --- | --- | --- |
| **Site type** | **Average site area** (mm^2^) | **Proportional area**  (average site area/average area of single site) | **Site count** | **Weighted site count, by area** (site count * proportional area) |
| Single | 312.199 | 1 | 125 | 125 |
| Double | 1405.8028 | 4.50290616 | 48 | 216.139496 |
| Triple | 1828.0052 | 5.85525642 | 9 | 52.6973078 |
| Bundled | 5499.7659 | 17.6162188 | 18 | 317.091939 |
| Total |  |  | 200 | 710.928742 |
